# Nutritional status and clinical outcome of children on continuous renal replacement therapy: a prospective observational study

**DOI:** 10.1186/1471-2369-13-125

**Published:** 2012-09-27

**Authors:** Ana Castillo, Maria J Santiago, Jesús López-Herce, Sandra Montoro, Jorge López, Amaya Bustinza, Ramón Moral, Jose M Bellón

**Affiliations:** 1 Pediatric Intensive Care Department Hospital General Universitario Gregorio Marañón, Universidad Complutense, Madrid, Spain; 2 Statistics, Preventive Medicine and Quality Service Hospital General Universitario Gregorio Marañón, Universidad Complutense, Madrid, Spain; 3 Pediatric Intensive Care Service, Hospital General Universitario Gregorio Marañón, Dr Castelo 47, Madrid, Spain

## Abstract

**Background:**

No studies on continuous renal replacement therapy (CRRT) have analyzed nutritional status in children. The objective of this study was to assess the association between mortality and nutritional status of children receiving CRRT.

**Methods:**

Prospective observational study to analyze the nutritional status of children receiving CRRT and its association with mortality. The variables recorded were age, weight, sex, diagnosis, albumin, creatinine, urea, uric acid, severity of illness scores, CRRT-related complications, duration of admission to the pediatric intensive care unit, and mortality.

**Results:**

The sample comprised 174 critically ill children on CRRT. The median weight of the patients was 10 kg, 35% were under percentile (P) 3, and 56% had a weight/P50 ratio of less than 0.85. Only two patients were above P95. The mean age for patients under P3 was significantly lower than that of the other patients (p = 0.03). The incidence of weight under P3 was greater in younger children (p = 0.007) and in cardiac patients and in those who had previous chronic renal insufficiency (p = 0.047). The mortality analysis did not include patients with pre-existing renal disease. Mortality was 38.9%. Mortality for patients with weight < P3 was greater than that of children with weight > P3 (51% vs 33%; p = 0.037). In the univariate and multivariate logistic regression analyses, the only factor associated with mortality was protein-energy wasting (malnutrition) (OR, 2.11; 95% CI, 1.067-4.173; p = 0.032).

**Conclusions:**

The frequency of protein-energy wasting in children who require CRRT is high, and the frequency of obesity is low. Protein-energy wasting is more frequent in children with previous end-stage renal disease and heart disease. Underweight children present a higher mortality rate than patients with normal body weight.

## Background

Many critically ill children present some degree of malnutrition, better known as protein-energy wasting, which impairs their response to disease and increases their susceptibility to infection and multi-organ failure, thus leading to a substantial rise in morbidity and mortality [1,2]. Protein-energy wasting can be acute as a result of the illness causing admission to the intensive care unit and chronic due to an underlying disease.

Children with acute kidney injury (AKI), especially those who need continuous renal replacement therapy (CRRT), have specific nutritional needs and often receive an excessive supply of carbohydrate and insufficient protein [3]. Studies in adults with chronic renal insufficiency reported an association between nutritional status and mortality, and some have shown that patients with a higher body mass index (BMI) may have better survival [4].

Several authors have assessed the impact of protein-energy wasting on long-term outcomes in children with end-stage renal disease (ESRD) receiving dialysis. Lower height and growth rate implies an increased mortality risk [5]. High and low BMI and low serum albumin are also associated with higher mortality [6].

Few authors have evaluated the nutritional status of patients with AKI and its association with clinical course. In a recent study of adults receiving CRRT, 4% were underweight, 38.4% overweight, and 21.6% obese [7]. The incidence of AKI increased in obese patients compared with normal-weight patients and those who were malnourished. Nevertheless, the obese patients receiving CRRT had a greater probability of survival than the malnourished or morbidly obese ones [7].

We performed the first study to assess the association between mortality and the nutritional status of children receiving CRRT.

## Methods

We analyzed nutritional status using a prospective observational registry including all critically ill children receiving CRRT who were admitted to the pediatric intensive care unit (PICU) between 1st January 1996 and 1st June 2009 [8]. The Gregorio Marañón University Hospital Review Board approved the study, and, at admission, parents gave their written informed consent for the children to participate in the study.

Most patients were weighed at admission. The scale used depended on the patient’s age. In the remaining patients, the parents were asked about the most recent weight and, if no recent weight was available, they were weighed in the PICU. The variables recorded prospectively at baseline were age, weight, sex, diagnosis, albumin, creatinine, urea, and uric acid. In addition, we recorded three severity of illness scores, namely, the Pediatric Risk of Mortality (PRISM II) score [9], the Pediatric Index of Mortality (PIM II) score [10], and the Pediatric Logistic Organ Dysfunction (PELOD) score [11], as well as mortality and CRRT-related complications (complications of catheterization [catheter-associated or other], hemorrhage, hypotension on connection to the filter, and electrolyte disturbances).

Weight percentiles and the real weight/P50 ratio were calculated according to local references [12,13]. Weight under percentile 3 (<P3) was considered protein-energy wasting or malnutrition. We also studied the real weight/P50 ratio using Waterlow’s modified criterion for acute protein-energy wasting, in which a ratio less than 0.85 was considered abnormal [14].

The statistical analysis was performed using SPSS version 16.0 (SPSS Inc., Chicago, Illinois, USA). The chi-squared test, Fisher exact test, and Mann–Whitney test were used to compare the qualitative and quantitative variables. Significance was taken as a p value less than 0.05. Univariate and multivariate logistic regression analyses were performed to analyze the influence of each factor on mortality. Variables with p < 0.1 were included in the multivariate analysis.

## Results

The study sample comprised 174 patients who had undergone CRRT. Of these, 97 patients (55.7%) had congenital heart disease and 81/97 (83.5%) had undergone heart procedures (14 heart recipients). Baseline characteristics of the patients and CCRT are shown in Tables 1 and 2. Mean age was 52.3 (SD 63.8) months; 76 (43.7%) were under one year old and 105 were male (60.3%). Mean weight was 17.7 kg (median 10 kg; IQR, 5-21.3) and 22% weighed less than 5 kg. Sixty-one patients (35%) were under P3 and 97 (56%) had a real weight/P50 ratio of less than 0.85.

**Table 1:** Baseline characteristics of the cohort

	**Median (P50)**	**IQR (P25-75)**
**Age (months)**	18.5	4.0-81.8
**Weight (kg)**	10.0	5.0-21.3
**PRISM score**	13.0	10.0-20.0
**PRISM (mortality percentage)**	21.7	7.1-52.8
**PIM score**	–2.2	–3.2 to 0.8
**PIM (mortality percentage)**	3.1	0.3-17.2
**PELOD score**	20.0	11.0-22.0
**PELOD (mortality percentage)**	18.5%	1.4-26.1
**Number of organ failures**	3.0	2.0-3.0
**Lactate (mmol/L)**	1.9	1.1-3.4
**Initial albumin (g/dL)**	3.0	2.4-3.4
**Initial creatinine (mg/dL)**	1.0	0.6-1.9
**Initial urea (mg/dL)**	63.0	44.5-106.5

PRISM, Pediatric Risk of Mortality (PRISM II); PIM, Pediatric Index of Mortality; PELOD, Pediatric Logistic Organ Dysfunction.

**Table 2:** Characteristics of CRRT

	**Median (P50)**	**IQR (P25-75)**
**Blood flow (ml/minute)**	50.0	30.0-85.0
**Ultrafiltrate (ml/kg/hour)**	6.4	3.3-11.1
**Reposition (ml/hour)**	200.0	100.0-400.0
**Dialysis (ml/hour)**	450	250-600
**Number of filters**	3.0	1.0-6.0
**Total duration of CRRT (hours)**	92.5	39.8-248.5

CRRT: continuous renal replacement therapy.

No differences in the frequency of protein-energy wasting were detected between sexes (weight < P3: male 36.2%, female 33.3% [p = 0.747]; real weight/P50 ratio <0.85: male 61%, female 47.8% [p = 0.118]).

The average age of the patients weighing under P3 was 34 (SD 56.5) months, significantly lower than the other children (62 [SD 65.7]) months (p = 0.03). The mean age of patients with a real weight/percentile 50 ratio <0.85 was 49.4 (SD 62.6) months. These patients were younger than those with a real weight/P50 ratio greater than 0.85; however, the differences did not reach statistical significance (p = 0.757).

When patients were classified into 4 age sub-groups, the incidence of weight under P3 was greater in the younger groups. A significant difference between those under 12 months and those over 5 years was observed (p = 0.007) (Table 3). However, these differences were not seen on testing the real weight/P50 ratio.

**Table 3:** Frequency of protein-energy wasting according to age group

**Age**	**<12 months**	**1-5 years**	**5-10 years**	**>10 years**	**p value**
Weight < P3	35 (46.1%)	16 (39%)	4 (14.8%)	6 (20%)	0.007
Ratio* <0.85	43 (56.6%)	26 (63.4%)	13 (48.1%)	15 (50%)	0.566

* Real weight/P50 ratio.

In terms of diagnosis, the frequency of weight under < P3 was significantly greater in patients with heart disease and in those who had already had previous chronic renal disease (p = 0.047). Although the incidence of protein-energy wasting was also greater in these groups when the analysis was performed using the real weight/P50 ratio, this difference did not reach statistical significance (p = 0.092) (Table 4).

**Table 4:** Frequency of protein-energy wasting in relation to diagnosis

**Diagnosis**	**Weight < P3**	Ratio* <0.85
Heart disease	39 (40.2%)	57 (58.8%)
Sepsis	8 (23.5%)	17 (50%)
ESRD	10 (59.9%)	14 (82.4%)
Other (inborn errors of metabolism, abdominal surgery, hemolytic uremic syndrome, tumor lysis syndrome)	4 (15.3%)	9 (34.6%)
p value	0.047	0.092

* Real weight/P50 ratio.

ESRD, end-stage renal disease.

No significant differences were observed in the PIM, PRISM, or PELOD scores between patients weighing over or under P3 and those with a real weight/P50 ratio over or under 0.85 (Table 5).

**Table 5:** Relation of body weight to mortality risk

	**PRISM mortality risk,%**	**PIM mortality risk,%**	**PELOD mortality risk,%**
Weight < P3	20.6	6.5	17.9
Weight > P3	19.9	12.9	24.1
p value	0.601	0.139	0.190
Ratio* <0.85	20.6	9.6	23.6
Ratio* >0.85	19.7	12.2	20.3
p value	0.724	0.877	0.929

PRISM, Pediatric Risk of Mortality (PRISM II); PIM, Pediatric Index of Mortality; PELOD, Pediatric Logistic Organ Dysfunction.

* Real weight/P50.

The incidence of hypoalbuminemia (defined as ≤2.5 g/dl) at the beginning of CRRT was 28%, with no significant differences between underweight patients and the remaining patients. After hemofiltration, the incidence of hypoalbuminemia diminished by 7%.

No differences were detected in the frequency of complications of CRRT associated with nutrition status. The most frequent complications were hemorrhage and hypotension at the beginning of the procedure (hemorrhage weight < P3 was 13.1%, weight > P3 was 9% [p = 0.440]; hypotension weight < P3 was 37.7%, weight > P3 was 27% [p = 0.169]).

No differences were found in the creatinine, urea, and uric acid values before CRRT in relation to the weight percentile or to the real weight/P50 ratio (data not shown); however, when we removed patients with pre-existing renal disease, we found differences in the remaining patients. Initial and 24-hour creatinine and urea were lower in patients with weight < P3 (p < 0.02).

The median duration of treatment was 78 hours (IQR, 40-186) in the < P3 weight group compared with 94.5 hours (IQR, 36-273) in the > P3 group (p = 0.309).

Global mortality was 35.6% (62 patients). The causes of death are detailed in Table 6. The mortality analysis did not include patients with pre-existing renal disease. Mortality in the remaining patients was 38.9%. Mortality in AKI patients with weight < P3 was greater than that of the children with weight > P3 (51% versus 33%; p = 0.037).

**Table 6:** Cause of death

**Cause**	**Frequency**	**Percentage**
Multi-organ failure	34	54.8
Cardiac shock	16	25.8
Brain death	6	9.7
Withdrawal of care	3	4.8
Respiratory insufficiency	2	3.2
Tumor recurrence	1	1.6
Total	62	100

No statistical differences in mortality were detected between patients with a real weight/P50 ratio <0.85 (42.2%) and those with a ratio >0.85 (35.1%) (p = 0.414).

In the univariate and multivariate logistic regression analyses, the only factor that was associated with mortality was protein-energy wasting (OR, 2.11; 95% CI, 1.067-4.173; p = 0.032).

In the multivariate logistic regression analysis, heart disease was not a confounding factor.

Overall, 23% of patients were over P50 and only 2 patients were over P95; therefore, it was not possible to study the association between obesity and mortality.

Mortality in children with hypoalbuminemia <2.5 g/dl at the beginning of CRRT was higher (46.7%) than the that of the other patients (33.8%), although the difference was not statistically significant (p = 0.215). The difference was significant in the patients who presented hypoalbuminemia at the end of treatment (83.3%) (p = 0.018).

## Discussion

Our study is the first to analyze nutritional status in critically ill children receiving CRRT. In adults with acute renal failure receiving CRRT, the percentage of malnutrition is low—4% in a recent study [7]—and the percentage of obesity is high. Nevertheless, the incidence of obesity in children with AKI in our study was very low, whereas the percentage of malnutrition was high (35%); the highest incidence of weight under P3 was observed among the youngest children. Children with previous chronic renal insufficiency and heart disease had the highest incidence of malnutrition. The prevalence of protein-energy wasting among children with chronic renal insufficiency rose from 53% to 64% depending on the definition considered [14].

Twenty-five percent of children with congenital heart disease are < P3 [15]. However, among hospitalized children with congenital heart disease, acute and chronic protein-energy wasting occurred in 33% and 64% of the patients [16].

Furthermore, cardiac surgery predisposes to the development of AKI secondary to low cardiac output, hemolysis after surgery, and infectious complications. Therefore, according to our study, children with heart disease present a higher incidence of malnutrition than other patients.

We found no association between mortality risk scores at the beginning of CRRT and nutritional status. We calculated three of the most widely used prognostic scores in critically ill children, which provide data for a number of clinical and analytical parameters. No differences in acute abnormalities of these parameters were observed between normal-weight and underweight children. However, nutritional status could affect subsequent clinical course, and these scores do not take into account nutritional factors.

In adults with AKI receiving CRRT, mortality rates follow a U-shaped pattern in which undernourished patients and morbidly obese patients are at the highest risk [7]. The anthropometric indicators with the greatest value for predicting mortality in these patients are forearm circumference, tricipital fold, and percentage of body fat [17,18].

In pediatric patients undergoing CRRT, the principal prognostic factors are hemodynamic abnormalities, disease severity, multi-organ failure, and previous positive balance at the beginning of the procedure [8,9,19].

In our study, mortality was greater in children whose weight was < P3 (42.6%) than children with a normal body weight (31.8%). Although considerable, the difference did not reach statistical significance. It was not possible to analyze the association between mortality and obesity, owing to the low number of children with obesity.

As for complications, obese adults required a longer duration of mechanical ventilation and a greater total number of days of hospitalization [7]. We found that children with malnutrition presented neither a greater incidence of complications nor longer duration of treatment than patients with normal body weight.

Several parameters have been recommended for monitoring the nutritional status of children with chronic renal insufficiency, such as serum albumin, height, weight, mid-arm circumference, skin fold thickness, and head circumference [20]. Some of these parameters (eg, height and head circumference) are useful in the case of prolonged malnutrition, although their usefulness is minimal in the critically ill child. Furthermore, the folds and circumferences of the arm are not useful in the evaluation of nutritional status in the critically ill child, because they could be altered by hydration status [21]. Therefore, nutritional status in critically ill children is evaluated mainly using anthropometric measures (weight) and biochemical analysis [21], although weight is also altered by hydration status.

We found that children with hypoalbuminemia presented higher mortality, although the difference was not statistically significant. Hypoalbuminemia has been associated with increased mortality in critically ill children [22]. Wong et al. found that children with ESRD and serum albumin <3.5 g/dl exhibited a 90% greater risk of death than patients with serum albumin >3.5 [6]. Nevertheless, albumin is not a good parameter for assessing malnutrition in critical patients because of its long half-life. Hypoalbuminemia in patients with renal failure is caused by a combination of reduced synthesis and increased degradation of albumin [23]. Moreover, serum albumin is affected not only by nutritional status, but also by fluid status, inflammation, and liver abnormalities [24,25]. Serum albumin has been shown to be a poor marker of nutritional status in severely malnourished children receiving hemodialysis [26,27].

Creatinine production depends on muscle mass and is age- and sex-related. We found that the creatinine and urea values were lower in underweight patients both before starting CRRT and during the first 24 hours.

Our study presents several limitations. The most important limitation is that body weight is not an ideal method for measuring the nutritional status of critically ill patients. Body weight is not available for all patients, can be affected by hydration status, cannot discriminate between acute and chronic malnutrition, and does not enable us to evaluate the evolution of nutritional status. Nevertheless, despite these limitations, body weight is the most widely used parameter for determining initial nutritional status in critically ill patients [1,14,28].

BMI was not taken into account, because height was not recorded in all patients. Other parameters such as prealbumin and transferrin or retinol-binding protein may prove more useful than albumin when evaluating protein nutritional status in the critically ill patient [21,29,30]. On the other hand, a high percentage of patients in our study underwent cardiac surgery; therefore, our results may not be representative of all critically ill children who require CRRT.

Although a high percentage of children receiving CRRT tolerate enteral nutrition [31], many receive insufficient calories and protein [3]. We did not analyze the effect of nutrition during admission to the PICU.

## Conclusions

Critically ill children who need CRRT present a high incidence of malnutrition, which is greater than that of adults. In contrast, the incidence of obesity is very low. Protein-energy wasting is more frequent in the youngest children, in patients with chronic renal insufficiency, and in patients with heart disease. Mortality is higher in malnourished children requiring CRRT than in those with normal body weight, although the difference is not statistically significant. Studies analyzing the effect of nutrition on the nutritional status and outcome of CRRT patients are necessary.

## Abbreviations

AKI: Acute Kidney Injury; CRRT: Continuous Renal Replacement Therapy; ESRD: End-stage Renal Disease; PIM: Paediatric Index of Mortality; PRISM: Pediatric Risk of Mortality; PELOD: Pediatric Logistic Organ Dysfunction.

## Competing interests

The authors declare no competing of interest.

## Author contributions

AC, SM and, J L contributed to the acquisition, analysis, and interpretation of data and gave their final approval of the manuscript. MJS and J L-H contributed to design of the study, the acquisition, analysis and interpretation of data, drafting of the manuscript and gave the final approval. AB and RM contributed in the analysis and interpretation of data, drafting of the manuscript and gave the final approval. JMB contributed in the acquisition, analysis and interpretation of data and gave the final approval.

## Pre-publication history

The pre-publication history for this paper can be accessed here:

http://www.biomedcentral.com/1471-2369/13/125/prepub
